# Bengamide Analogues Show A Potent Antitumor Activity against Colon Cancer Cells: A Preliminary Study

**DOI:** 10.3390/md18050240

**Published:** 2020-05-02

**Authors:** Beatriz García-Pinel, Cristina Porras-Alcalá, Laura Cabeza, Raul Ortiz, José Prados, Consolación Melguizo, Iván Cheng-Sánchez, Juan Manuel López-Romero, Francisco Sarabia

**Affiliations:** 1 Institute of Biopathology and Regenerative Medicine (IBIMER), Center of Biomedical Research (CIBM), University of Granada, 18100 Granada, Spain; beatrizgarnel@ugr.es (B.G.-P.); roquesa@ugr.es (R.O.); jcprados@ugr.es (J.P.); melguizo@ugr.es (C.M.); 2 Department of Anatomy and Embriology, Faculty of Medicine, University of Granada, 18071 Granada, Spain; 3 Instituto de Investigación Biosanitaria ibs, Granada, 18012 Granada, Spain; 4 Department of Organic Chemistry, Faculty of Sciences, University of Malaga, 29071 Málaga, Spain; cporrasalcala@gmail.com (C.P.-A.); cheng@uma.es (I.C.-S.)

**Keywords:** bengamides, analogues, synthesis, antitumor agents, colorectal cancer

## Abstract

The limited success and side effects of the current chemotherapeutic strategies against colorectal cancer (CRC), the third most common cancer worldwide, demand an assay with new drugs. The prominent antitumor activities displayed by the bengamides (Ben), a family of natural products isolated from marine sponges of the *Jaspidae* family, were explored and investigated as a new option to improve CRC treatment. To this end, two potent bengamide analogues, Ben I (**5**) and Ben V (**10**), were selected for this study, for which they were synthesized according to a new synthetic strategy recently developed in our laboratories. Their antitumor effects were analyzed in human and mouse colon cell lines, using cell cycle analysis and antiproliferative assays. In addition, the toxicity of the selected analogues was tested in human blood cells. These biological studies revealed that Ben I and V produced a significant decrease in CRC cell proliferation and induced a significant cell cycle alteration with a greater antiproliferative effect on tumor cell lines than normal cells. Interestingly, no toxicity effects were detected in blood cells for both compounds. All these biological results render the bengamide analogues Ben I and Ben V as promising antitumoral agents for the treatment of CRC.

## 1. Introduction

Colorectal cancer (CRC), the third most common cancer worldwide [1], increased its incidence over the last year as a result of the population aging, sedentary lifestyle and nutrition [2]. Surgery is the treatment of choice for patients with non-metastatic CRC [3], but a high proportion of them are diagnosed in stage IV with distant metastasis (advanced cancer), thus requiring chemotherapy. Currently, CRC chemotherapy is essentially based on 5-fluorouracil (5-FU), oxaliplatin (OXA) and irinotecan (IRI). Furthermore, combined therapy is commonly used as a first-line treatment as it offers better response rates and progression-free survival compared to monotherapy. Moreover, monoclonal antibodies and multi-kinase inhibitors have been introduced in CRC treatment (cetuximab, panitumumab, bevacizumab) [4,5]. However, despite advances in treatment, CRC has moved from fourth place among cancers with the highest mortality to second place, after lung cancer [6,7]. In addition, these therapies are often associated with major side effects and specific disorders, such as hand-foot syndrome in the case of 5-FU [8], or neuro-, oto- and nephrotoxicity in the case of OXA [9] and with the appearance of the multidrug resistance (MDR) phenotype [10]. The increase in the mortality rate and the development of side effect and drug resistance phenomena makes it necessary to develop new treatments and strategies that improve the results obtained with current first-line treatments [11,12]. In this context, given the outstanding antitumor properties of the bengamides, a family of natural products produced by marine sponges of the *Jaspidae* family, we decided to explore the potential of these compounds for the treatment of colon cancer.

**Figure 1:**
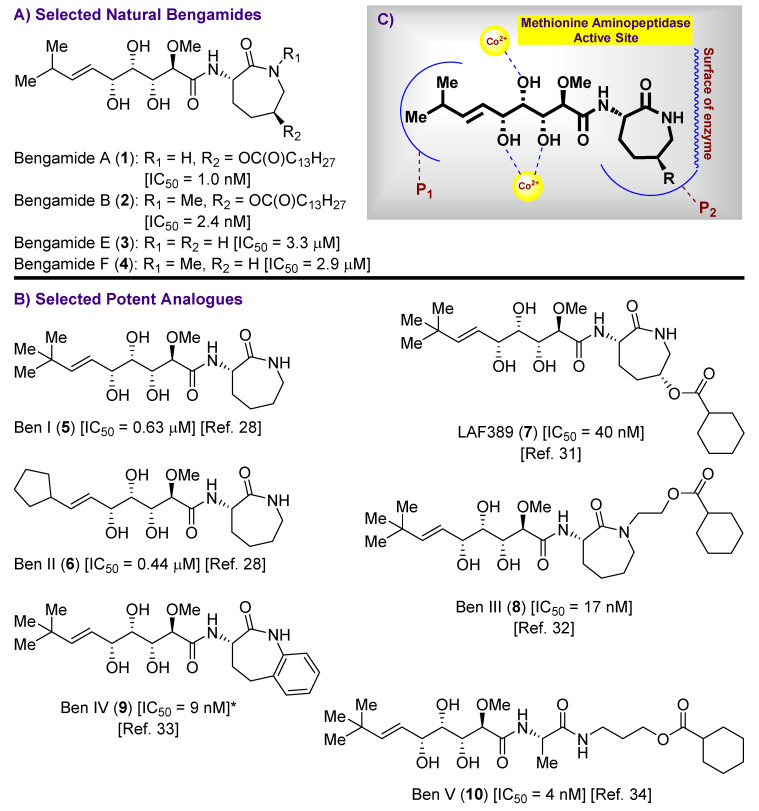
(**A**) Molecular structures of selected natural bengamides (**1**–**4**); (**B**) Representative bengamide analogues (**5**–**10**) and their cytotoxicities against MDA-MB-435 human breast cancer cells (* For analogue **9**, the value of IC_50_ is against the cancer cell line A549); (**C**) Mode of interaction of the bengamides at the active site of the methionine aminopeptidases.

The bengamides (See Figure 1 for representative members **1**–**4**) were discovered in 1986 [13] and elicited great biological and chemical interest in virtue to their prominent antitumor, antihelmintic and antibiotic properties [14]. Particularly striking are their antiproliferative activities, displaying cytotoxicities in the 1.0 nM–3.3 μM range for the IC_50_ values against human breast MDA-MB-435 carcinoma cells and producing the arrest of the cells at the G_1_ and G_2_M phases of the cell cycle [15]. Proteomic studies revealed that the bengamides inhibited both methionine aminopeptidases types 1 and 2 (MetAPs 1 and 2), enzymes responsible of the cleavage of the *N*-terminal initiator methionine residue during protein synthesis [16]. These biological findings were supported by the isolation and subsequent X-ray analysis of the complex enzyme-bengamide [17], and more recently, by the reported X-ray structures of four bengamide analogues in complex with *Hs*MetAP1 in the Mn (II) form [18]. All these X-ray structures revealed the mode of interaction of these bioactive compounds at the active site of the methionine aminopeptidases, according to which a critical dinuclear metal center placed as a deep invagination in the surface of the enzyme is coordinated with the hydroxyl groups at C3, C4 and C5. On the other hand, a hydrophobic pocket P1, which contains the residues Phe-219, His-382 and Ala-414, in the innermost portion of the active-site, interacted with the terminal alkyl group of the olefin, while a pocket P2, formed at the solvent-exposed surface by the residues of Leu-328, Phe-366 and His-231, holds the caprolactam ring (Figure 1).

Interestingly, the enzyme MetAP2 is the biological target of the very well-known anti-angiogenic compounds fumagillin and ovalicin [19,20], which render bengamides as new promising anticancer leads with potential anti-angiogenic properties. Further biological investigations in this field were accomplished to elucidate the key protein or proteins that could be affected by the inhibition of methionine aminopeptidases and, as a consequence, trigger the observed antitumor effect. As a result of these investigations, the proto-oncogene c-Src, involved in the development, growth, progression and metastasis of a number of human cancers [21], was identified and validated as a substrate for both MetAP1 and MetAP2 in vivo and in vitro. Thus, in this research, it was proved that the inhibition of MetAPs by the nonselective inhibitor bengamide A (**1**) altered the subcellular distribution of this proto-oncogene [22]. This alteration significantly decreased its tyrosine kinase activity and caused a remarkable delay in cell-cycle progression. Therefore, these results establish a link between c-Src and MetAP and suggest that inhibition of MetAPs could indirectly impair the functions of c-Src and probably other oncogenes that are essential for tumor growth. Additional biological studies achieved by Crews and coworkers led to the disclosure that the bengamides also exhibit an inhibitory activity against the NF-κB (nuclear factor kappa B), which could be also related with their observed antitumor activities due to the close relationship between tumorigenesis and inflammation [23]. This inhibition activity has been recently associated to the antiviral activity against HIV-1 observed by bengamide A [24]. On the other hand, the ability of the bengamides to inhibit MetAPs from *Mycobacterium tuberculosis* has been exploited in the treatment of tuberculosis [25,26].

These striking biological activities, together with their unique molecular structures, have prompted an intense synthetic activity directed towards the total syntheses of the natural products and analogues thereof analogues in order to identify and develop new chemical entities with improved antitumor and pharmacokinetic properties with respect to the natural counterparts [14,27]. The biological evaluations of all these analogues have allowed for the establishment of an extensive structure-activity relationship, revealing the following key structural conclusions: (a) the importance of the substituent at the terminal olefinic position for the antiproliferative activity, as demonstrated with the bengamide E analogues **5** and **6** [28]; (b) the essential role of the polyketide fragment, whose hydroxyl groups and stereochemistry can not be modified to maintain their antitumor activities [29,30]; and (c) the beneficial impact of the modification of the caprolactam fragment in their antitumor properties as demonstrated with the representative analogues **7**–**10** [31,32,33,34] (Figure 1). Particularly promising was the bengamide A analogue **7**, known as LAF389, which, developed by Novartis, was considered as a clinical candidate [35]. However, its poor pharmacokinetic properties hampered further clinical development. Similarly interesting were the ring-opened bengamides, which were identified as highly potent antitumor analogues against MDA-MB-435 and improved water solubilities. Among the analogues described of this series, the ring-opened bengamide **10**, described by Nan et al., was identified as the most potent bengamide analogue of the series, with an IC_50_ value of 4 nM against MDA-MB-435 human breast cancer cells [34]. 

Based on the excellent and promising antitumor properties of the bengamides, and more particularly, of some of their analogues, we decided to investigate the antitumor activities and the viability of selected analogues against CRC cell lines as a new alternative treatment of colon cancer. For this study, we selected the analogues **5** (Ben I) and **10** (Ben V), which display very potent antiproliferative activities against various tumor cell lines and suitable solubilities in water.

## 2. Results and Discussion

### 2.1. Synthesis of the Bengamide Analogues

**Scheme 1:**
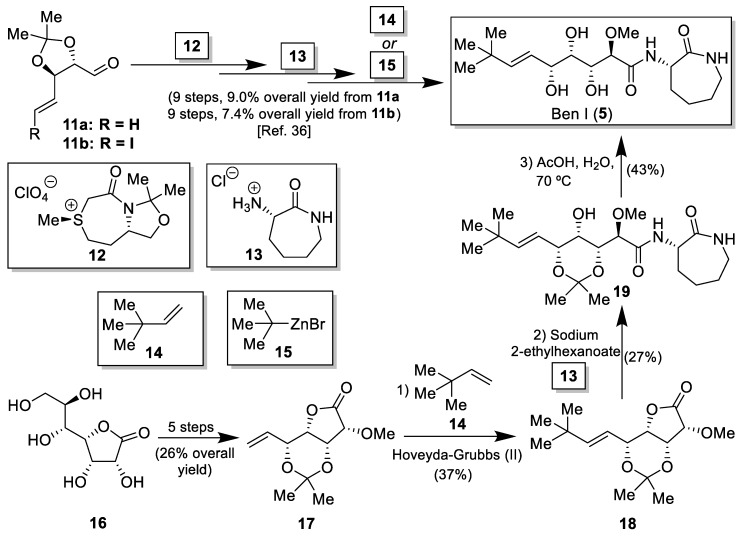
Synthesis of bengamide analogue **5** (Ben I).

The synthesis of the bengamide analogue **5** (Ben I) was reported earlier by us from aldehydes **11a** or **11b** in nine steps and in 9.0% and 7.4% overall yields, respectively, according to a new methodology of epoxidation based on the use of a new class of chiral sulfonium salts (compound **12**), combined with a key cross metathesis reaction, employing commercially available alkene **14** for **11a**, or a Negishi coupling with the organometallic derivative **15** for the case of **11b** as starting aldehyde [36]. This synthetic strategy proved to be efficient and flexible not only in providing access to the natural bengamides but also to an array of analogues modified at the terminal olefinic [28] and at C-2 positions [29]. Alternatively, in order to secure a shorter synthetic route to analogue **5**, the D-glucoheptono 1,4-lactone (**16**) was exploited as starting material, which was transformed into the advanced precursor **18** in six steps, through intermediate **17 [37,38]**. Thus, the reaction of **18** with the commercially available ε-aminocaprolactam **13** under basic conditions, by treatment with sodium 2-ethylhexanoate in THF, provided the coupling product **19**, albeit in a lower yield (27%), with respect to that reported in the literature (75%) [39]. Acidic hydrolysis of **19** afforded the targeted analogue **5** in a modest 43% yield (Scheme 1). 

**Scheme 2:**
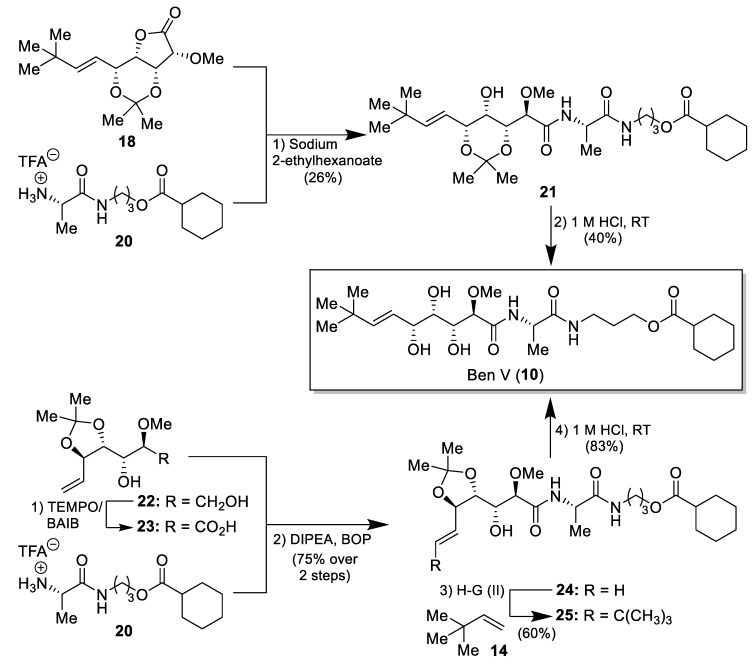
Synthesis of bengamide analogue **10** (Ben V).

On the other hand, the synthesis of the ring-opened bengamide **10** (Ben V), described by Nan and coworkers as one of the most potent bengamide analogues described so far [34], was accomplished in a similar manner by coupling of lactone **18** with amino acid derivative **20**. The resulting coupling product **21**, obtained in a poor 26% yield, was then subjected to acidic hydrolysis to furnish the coveted bengamide analogue **10** (Ben V). As for the coupling of **18** with **13**, described before for the synthesis of analogue **5**, the key coupling reaction proceeded in a lower yield compared with the reported by Nan et al., who obtained product **21** in a 55% yield under identical reaction conditions [34] (Scheme 2). Aiming to improve the yield of this coupling reaction, either for the synthesis of **21** as for **19**, extensive experimentation was carried out by modification of the reaction conditions (for example, sodium 2-ethylhexanoate as base in THF at 50 °C, *i*-PrOH at 50 °C or reflux) and the employed base, such as DIPEA, Et_3_N or DBU, under different conditions (THF at 50 °C, *i*-PrOH at 80 °C). To our dismay, in all the cases, we were not able to improve the yields for the coupling products (**19** or **21**), which were obtained in the same low ranges as before (15–25% yields), recovering unreactive lactone **18** together with some degradation products. In light of these discouraging results, we decided to carry out a direct coupling of amino acid derivative **20** with the acid **23**, resulting from the selective oxidation of diol **22**, which was readily prepared from D-lactic acid, according to the procedure developed in our laboratories [36]. For this reaction, the coupling reagent (benzotriazol-1-yloxy) tris(dimethylamino)phosphonium hexafluorophosphate (BOP) was chosen as most suitable, and the result was the obtention of the product **24** in a good 75% yield. The coupling reaction was then followed by the assembly of both olefins, compounds **24** and **14**, for which a cross metathesis reaction mediated by the Hoveyda–Grubbs second generation catalyst was initially attempted. To our delight, the cross-metathesis reaction proceeded smoothly to afford the product **25** in a reasonable 60% yield. Final acetal deprotection of compound **25** provided the targeted analogue **10** in an 83% yield (Scheme 2). With both bengamide analogues in hand, we then proceeded with the biological studies as described in the following sections.

### 2.2. In Vitro Antiproliferative Assays

Both Ben I and Ben V (analogues **5** and **10**, respectively) IC_50_ (µM) were determined in all cell lines, finding significant differences between both compounds (*p* < 0.05) except in CCD18 (*p* > 0.05) (Table 1). 

**Figure 2:**
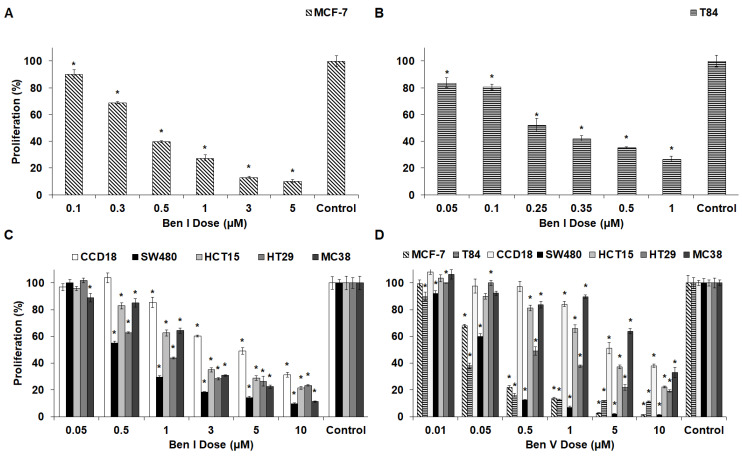
Antiproliferative assays of the Ben I and Ben V compounds. Ben I’s effect after 72 h of treatment in (**A**) MCF7; (**B**) T84 and (**C**) rest of the cell lines. Ben V’s effect after 72 h of treatment in all cell lines (**D**). Data represent the mean values ± SD of triplicate cultures. * Statiscally significant differences (*p* < 0.05) with the untreated control.

Both treatments produced significant tumor cell death (*p* < 0.05) and were especially effective on lines MCF7, T84 and SW480, the analogue Ben V being much more effective in all cases (Figure 2). As for HT29, the Ben V analogue is slightly more effective than the Ben I analogue (Figure 2C,D). Only in CCD18 normal colon cell line and in HCT15 resistant colon cancer cell line was Ben I analogue more effective than Ben V (Figure 2C,D). Similar results were found in MC38 mouse colon cancer cell line, in which Ben I clearly induced a greater effect than Ben V (Figure 2C,D). For Ben I, statistically significant differences (*p* < 0.05) were observed with respect to the control without treatment at all doses in cell lines MCF7, T84 and MC38, from the second dose in SW480, HCT15 and HT29, and from the third dose in CCD18. For Ben V, statistically significant differences were observed with respect to the control in all doses in cell lines T84, SW480 and HT29, from the second dose in MCF7, in MC38 and HCT15 from the third and in CCD18 from the fourth. Regarding their reference compound, both Ben I and Ben V produce a greater antiproliferative effect in most of the cell lines studied, except for MCF7 and MC38 lines (Table 1).

### 2.3. Blood Cell Cytotoxicity

**Figure 3:**
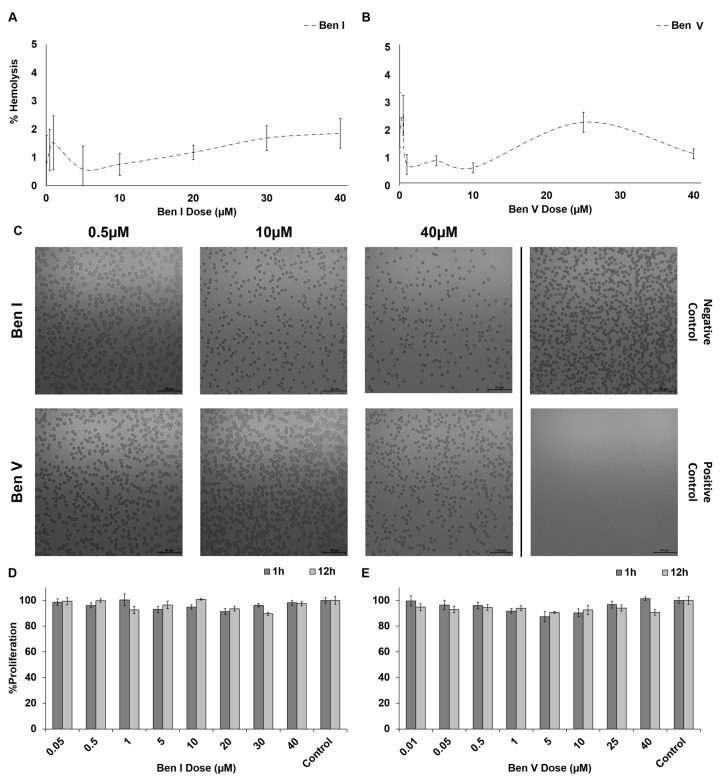
Blood cell cytotoxicity analysis for Ben I and Ben V analogues. Hemolysis test for Ben I (**A**) and Ben V (**B**). Optical microscopy analysis of erythrocytes after both treatments (different doses). Positive (20% Triton X-100) and negative (PBS) controls were included. Scale bar 50 µm (**C**). Cytotoxicity test in white blood cells (WBC) for Ben I (**D**) and Ben V (**E**). Data represent the mean values ± SD of triplicate experiment.

In order to evaluate the toxicity of both Ben I and Ben V compounds on blood cells, a hemolysis test was performed using human erythrocytes. As shown in Figure 3A,B, a very low level of hemolysis (around 2%) was detected at the highest doses of both Ben I and Ben V analogues, supporting their lack of hemotoxicity. In addition, none of these compounds caused erythrocytes agglutination or modification of their morphology (Figure 3C). On the other hand, an absence of toxicity in white blood cells (WBC) (viability ~ 100%) was detected for both Ben I and Ben V analogues after 1 and 12 h of exposure at all doses tested (Figure 3D,E).

### 2.4. Cell Cycle Analysis 

**Figure 4:**
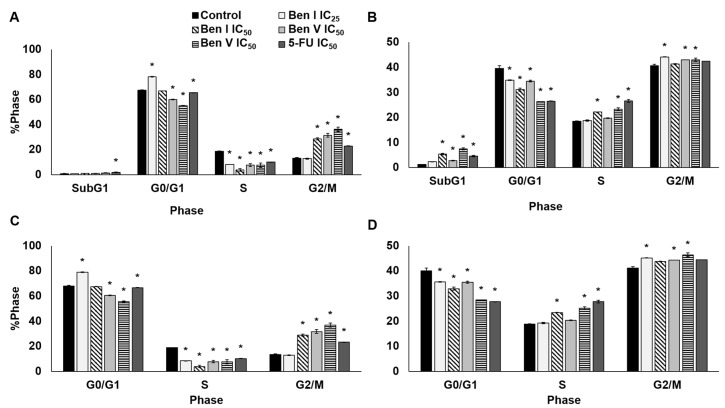
Cell cycle analysis after Ben I and Ben V exposure. Effect of Ben I, Ben V and 5-FU (positive control) in cell cycle of lines T84 (**A**) and CCD18 (**B**). Cell cycle analysis without consider SubG1 phase in T84 (**C**) and CCD18 (**D**). * Statiscally significant differences (*p* < 0.05) with the untreated control.

Cell cycle analysis showed a differential behavior when cells were treated with Ben I and Ben V (Figure 4). In fact, in T84 cells showed a statistically significant (*p* < 0.05) G2/M phase increase whith both treatments. However, this increase was much greater after Ben V exposure, including a slight reduction (*p* < 0.05) in the G0/G1 and S phases. No differences were observed with the control in the SubG1 phase for Ben I and Ben V (*p* > 0.05). By contrast, CCD18 normal colon cells showed a marked increase (*p* < 0.05) in the SubG1 and S phase with both Ben I and Ben V compounds (at doses of IC_50_), while a slight increase (*p* < 0.05) was observed in the G2/M phase, especially for Ben V.

## 3. Experimental Section

### 3.1. General Techniques 

All reactions were carried out under an argon atmosphere with dry, freshly distilled solvents under anhydrous conditions, unless using aqueous reagents or otherwise noted. All solvents used in reactions were dried and distilled using standard procedures. Tetrahydrofuran (THF) was distilled from sodium benzophenone, and methylene chloride (CH_2_Cl_2_) from calcium hydride. Yields refer to chromatographically and spectroscopically (^1^H NMR) homogeneous materials, unless otherwise stated. All solutions used in workup procedures were saturated unless otherwise noted. All reagents were purchased at highest commercial quality and used without further purification unless otherwise stated. All reactions were monitored by thin-layer chromatography (TLC) using 0.25 mm silica gel plates (60F-254) using UV light (254 nm) as visualizing agent and acidic ceric ammonium molybdate/ phosphomolybdic acid or potassium permanganate solutions and heat as developing agents. Flash column chromatography (FCC) was performed using silica gel (60 Å, particle size 230–400 mesh) under air pressure. All solvents used for chromatographic purifications were distilled prior to use. ^1^H and ^13^C NMR spectra were recorded on a Bruker DPX-400 MHz instrument (Fällanden, Switzerland) and calibrated using residual undeuterated solvent as an internal reference. Chemical shifts are reported in ppm with the resonance resulting from incomplete deuteration of the solvent as the internal standard (^13^CDCl_3_: 7.26 ppm, s and 77.0 ppm, t; ^13^CD_3_OD: 4.87 ppm, s, 3.31 ppm, quin and 49.1 ppm, sep; ^13^C_2_D_6_OS: 2.49 ppm, quin and 39.52 ppm, sep). Data were reported as follows: chemical shift δ/ppm (multiplicity, coupling constants *J* (Hz) and integration (^1^H only)). The following abbreviations were used to explain the multiplicities: s = singlet; d = doublet; t = triplet; q = quartet; quin = quintet; b = broad; m = multiplet or combination thereof. ^13^C signals are singles, unless otherwise stated. The corresponding NMR spectra are displayed in Supplementary Figures S2–S6. High-resolution mass spectrometry (HRMS) was performed on a Thermo Fisher Scientific H-ESI and APCI mass spectrometer (Waltham, MA, USA) in positive mode and using an ion trap (Orbitrap) as the mass analyzer type. HRMS signals were reported to four decimal places and are within ±5 ppm of theoretical values. 

### 3.2. Synthesis of the Bengamide Analogues

**Alkene 18.** Olefin **17** (220 mg, 1.0 mmol, 1.0 equiv) and Hoveyda–Grubbs 2nd generation catalyst (940 mg, 0.15 mmol, 0.15 equiv) were dissolved in degassed CH_2_Cl_2_ (10 mL). Then, 3,3-dimethylbut-1-ene (5 eqv, 5 mmol, 420 mg, 0.64 mL) was added dropwise at room temperature. The resulting mixture reaction was heated at 40 °C for 12 h. After this time, the solvent was removed in vacuo and the resulting crude product was purified by flash column chromatography (Silica gel, 100% hexanes → 40% EtOAc in hexanes) to obtain **17** (160 mg, 37%) as a brown solid: R*_f_*: 0.59 (silica gel, 80% EtOAc in hexanes); ^1^H NMR (400 MHz, CDCl_3_): δ 5.84 (d, *J* = 15.8 Hz, 1H), 5.58 (dd, *J* = 15.8, 7.6 Hz, 1H), 4.71 (dd, *J* = 3.8, 2.1 Hz, 1H), 4.45 (d, *J* = 8.9 Hz, 1H), 4.09 (d, *J* = 3.8 Hz, 1H), 3.99 (t, *J* = 2.0 Hz, 1H), 3.66 (s, *J* = 3.8 Hz, 3H), 1.53 (s, 3H), 1.49 (s, 3H), 1.03 (s, *J* = 2.1 Hz, 9H). 

**Precursor 19.** Alkene **18** (81 mg, 0.285 mmol, 1.0 equiv) and Ɛ-aminocaprolactam **13** (70.3 mg, 0.427 mmol, 1.5 equiv) were dissolved in anhydrous THF (15 mL). Then, sodium 2-ethylhexanoate (282 mg, 3.36 mmol, 6.0 equiv) was added and the resulting mixture was stirred at room temperature for 48 h. After this time, cyclohexane and water were added and the mixture was stirred for 30 min at room temperature. The aqueous phase was extracted with CH_2_Cl_2_ and the organic layer was washed with brine, dried over anhydrous MgSO_4_, filtered and solvents were removed in vacuo. The resulting crude product was purified by flash column chromatography (Silica gel, 40% EtOAc in hexanes → 5% MeOH in CH_2_Cl_2_) to provide compound **18** (32.5 mg, 27%) as a brown solid: R*_f_*: 0.50 (silica gel, 10% MeOH in CH_2_Cl_2_); ^1^H NMR (400 MHz, CDCl_3_) δ 7.56 (d, *J* = 6.5 Hz, 1H), 6.30 (dd, *J* = 11.2, 5.1 Hz, 1H), 5.77 (dd, *J* = 15.8, 0.9 Hz, 1H), 5.52 (dd, *J* = 15.8, 6.8 Hz, 1H), 4.63–4.55 (m, 1H), 4.29–4.24 (m, 1H), 4.06 (dd, *J* = 7.2, 1.1 Hz, 1H), 3.87 (d, *J* = 7.2 Hz, 1H), 3.50–3.49 (m, 1H), 3.47 (s, 3H), 3.30–3.22 (m, 2H), 2.89–2.83 (m, 1H), 2.13–2.03 (m, 2H), 1.89–1.79 (m, 3H), 1.57–1.51 (m, 1H), 1.46 (s, 3H), 1.44 (s, 3H), 1.38–1.30 (m, 2H), 1.02 (s, 9H); HRMS (H-ESI) *m/z*: [M + H]^+^ calculated for C_21_H_37_N_2_O_6_ 413.2652; found 413.2675.

**Bengamide analogue 5.** A 70% aqueous AcOH solution (5.85 mL) was added dropwise to a stirred solution of compound **19** (32.5 mg, 0.078 mmol, 1.0 equiv) in MeOH (2.5 mL). The resulting reaction mixture was heated at 70 °C for 2 h. After this time, the crude reaction was diluted with EtOAc and the excess of AcOH was quenched by addition of a saturated aqueous NaHCO_3_ solution. The aqueous phase was extracted with EtOAc and the combined organic layers were washed with brine, dried over anhydrous MgSO_4_, filtered and the solvent evaporated under reduced pressure. The residue was purified by flash column chromatography (Silica gel, 40% EtOAc in hexanes → 10% MeOH in CH_2_Cl_2_) to obtain bengamide analogue **5** (12.5 mg, 43%) as a colourless oil: R*_f_*: 0.1 (silica gel, 10% MeOH in CH_2_Cl_2_); ^1^H NMR (400 MHz, CDCl_3_) δ 7.98 (d, *J* = 6.4 Hz, 1H), 6.29 (t, *J* = 5.6 Hz, 1H), 5.82 (dd, *J* = 15.7, 0.9 Hz, 1H), 5.41 (dd, *J* = 15.7, 7.3 Hz, 1H), 4.54 (ddd, *J* = 11.3, 6.3, 1.2 Hz, 1H), 4.23 (dd, *J* = 6.6, 5.7 Hz, 1H), 3.84–3.76 (m, 2H), 3.60 (d, *J* = 4.8 Hz, 1H), 3.53 (s, 3H), 3.32–3.25 (m, 2H), 2.10–2.03 (m, 2H), 1.91–1.80 (m, 2H), 1.64–1.54 (m, 1H), 1.49–1.37 (m, 1H), 1.28–1.23 (m, 2H), 1.02 (s, 9H); HRMS (H-ESI) *m/z*: [M + H]^+^ calculated for C_18_H_33_N_2_O_6_ 373.2339; found 373.2341.

**Compound 21.** To a solution of lactone **18** (100 mg, 0.351 mmol, 1.0 equiv) and amino acid derivative **20** (195.4 mg, 0.53 mmol, 1.5 equiv) in anhydrous THF (12 mL) was treated with sodium 2-ethylhexanoate (232 mg, 1.14 mmol, 4.0 equiv) at room temperature for 4 days. After this time, cyclohexane and water were added and the mixture was stirred for 30 min at room temperature. The aqueous phase was then extracted with CH_2_Cl_2_ and the organic layer washed with brine, dried over anhydrous MgSO_4_, filtered and concentrated under reduced pressure. Purification by flash column chromatography (Silica gel, 40% EtOAc in hexanes → 5% MeOH in CH_2_Cl_2_) of the resulting crude product afforded compound **21** (53 mg, 26%) as a light yellow oil: R*_f_*: 0.20 (silica gel, 10% MeOH in DCM); ^1^H NMR (400 MHz, CDCl_3_) δ 6.90 (d, *J* = 8.3 Hz, 1H), 6.73 (t, *J* = 6.3 Hz, 1 H), 5.77 (d, *J* = 15.81H), 5.50 (dd, *J* = 15.8, 6.9 Hz, 1H), 4.52 (dq, *J* = 14.1, 6.9 Hz, 1H), 4.30–4.22 (m, 1H), 4.17 (dd, *J* = 6.0, 1.4 Hz, 1H), 4.13–4.03 (m, 3H), 3.94–3.90 (m, 1H), 3.51 (s, 3H), 3.28 (td, *J* = 13.4, 6.8 Hz, 2 H), 2.33–2.24 (m, 1H), 1.92–1.85 (m, 3 H), 1.85–1.80 (m, 2 H), 1.79–1.70 (m, 4 H), 1.67–1.60 (m, 2 H), 1.57–1.53 (m, 1 H), 1.48 (s, 3 H, overlapped), 1.47 (s, 3 H, overlapped), 1.41 (d, *J* = 7.1 Hz, 3 H), 1.01 (s, 9 H); HRMS (H-ESI) *m/z*: [M + H]^+^ calculated for C_28_H_49_N_2_O_8_ 541.3489; found 541.3504.

**Bengamide analogue 10.** HCl (1 M, 17 mL) was added to a stirred solution of compound **21** (53 mg, 0.098 mmol, 1.0 equiv) in THF (17 mL). The resulting reaction mixture was vigorously stirred for 4 h at room temperature. After this time, the reaction mixture was diluted with EtOAc and the excess of HCl was quenched by addition of a saturated aqueous NaHCO_3_ solution. The aqueous layer was extracted with AcOEt, the organic layer washed with brine, dried over anhydrous MgSO_4_, filtered and concentrated under reduced pressure. The resulting crude product was purified by flash column chromatography (Silica gel, 60% EtOAc in hexanes → 20% MeOH in CH_2_Cl_2_) to obtain bengamide analogue **10** (19.6 mg, 40%) as a colourless oil: R*_f_*: 0.1 (silica gel, 10% MeOH in DCM); ^1^H NMR (400 MHz, CDCl_3_): δ 7.13 (s, 1H), 6.79 (s, 1H), 5.84 (d, *J* = 15.7 Hz, 1H), 5.49 (dd, *J* = 15.6, 6.4 Hz, 1H), 4.55–4.40 (m, 2H), 4.27–4.18 (m, 1H), 4.10 (t, *J* = 6.5 Hz, 2H), 4.00 (s, 1H), 3.83 (d, *J* = 4.2 Hz, 1H), 3.69 (s, 1H), 3.50 (s, 3 H), 3.40–3.26 (m, 2H), 3.18 (dt, *J* = 13.5, 6.2 Hz, 1H), 2.35–2.24 (m, 2H), 1.95–1.70 (m, 8H), 1.45 (d, *J* = 7.2 Hz, 3H), 1.25 (s, 4H), 1.03 (s, *J* = 0.9 Hz, 9H); HRMS (H-ESI) *m/z*: [M + H]^+^ calculated for C_25_H_45_N_2_O_8_ 501.3176; found 501.3158.

**Carboxylic acid 23.** BAIB (196 mg, 0.61 mmol, 3.0 equiv) was added to a stirred solution of diol **22** (50 mg, 0.2 mmol, 1.0 equiv) in a 1:1 mixture of acetonitrile: water (2.0 mL). Then, 2,2,6,6-tetramethyl-1-piperidinyloxy (TEMPO) (9.4 mg, 0.06 mmol, 0.3 equiv) was added and the resulting reaction mixture was stirred for 12 h at room temperature. After this time, the reaction was quenched by addition of a saturated aqueous Na_2_S_2_O_3_ solution and the aqueous layer was extracted with AcOEt. The combined organic extracts were washed with brine, dried over anhydrous MgSO_4_, filtered and concentrated under reduced pressure to yield carboxylic acid **23** as a light yellow oil, which was used in the next step without any further purification. 

**Compound 24.** To a solution of crude acid **23** (23 mg, 0.07 mmol, 1.0 equiv) in DMF (1 mL) were added DIPEA (0.37 mL, 2.31 mmol, 3.3 equiv) and amino acid derivative **20** (40.7 mg, 0.11 mmol, 1.5 equiv). When the solution was homogeneous, BOP (37 mg, 0.08 mmol, 1.2 equiv) was added in one portion, and the reaction mixture was stirred for 12 h at room temperature. After this time, Et_2_O was added, and the organic phase was washed with a saturated aqueous NH_4_Cl solution. The organic layer was dried over anhydrous MgSO_4_, filtered and solvents were removed in vacuo. The resulting crude product was purified by flash column chromatography (Silica gel, 60% EtOAc in hexanes → 100% EtOAc) to obtain compound **24** (25 mg, 75% over 2 steps from **22**) as a white solid. R*_f_*: 0.49 (silica gel, 100% EtOAc); ^1^H NMR (400 MHz, CDCl_3_) δ 6.97 (d, *J* = 7.7 Hz, 1H), 6.83–6.72 (m, 1H), 5.94–5.82 (m, 1H), 5.47–5.26 (m, 2H), 4.55–4.36 (m, 2H), 4.23–4.07 (m, 3H), 3.94 (dd, *J* = 8.4, 2.5 Hz, 1H), 3.71 (d, *J* = 2.7 Hz, 1H), 3.46 (s, *J* = 12.5 Hz, 3H), 3.34–3.22 (m, 2H), 2.34–2.26 (m, 1H), 1.92–1.72 (m, 8H), 1.45 (s, 3H), 1.43 (s, 3H). ^13^C NMR (101 MHz, CDCl_3_) δ 171.79, 171.02, 135.15, 130.08, 128.29, 119.66, 109.57, 81.97, 79.31, 78.67, 70.78, 61.30, 59.21, 48.62, 43.22, 36.23, 29.71, 29.04, 28.59, 27.16, 26.88, 25.71, 25.41, 17.87; HRMS (H-ESI) *m/z*: [M + H]^+^ calculated for C_24_H_41_N_2_O_8_ 485.2863; found 485.2872.

**Compound 25.** Olefin **24** (25 mg, 0.05 mmol, 1 equiv) and Hoveyda–Grubbs 2nd generation catalyst (4.7 mg, 0.008 mmol, 0.15 equiv) were dissolved in degassed CH_2_Cl_2_ (3 mL). Then, 3,3-dimethylbut-1-ene **14** (12.6 mg, 0.15 mmol, 0.19 mL, 3 equiv) was added dropwise at room temperature. The resulting mixture reaction was heated at 40 °C for 48 h. After this time, the solvent was removed in vacuo and the resulting crude product was purified by flash column chromatography (Silica gel, 20% EtOAc in hexanes → 60% EtOAc in hexanes) to obtain **25** (16 mg, 60%) as a brown solid: R*_f_*: 0.60 (silica gel, 100% EtOAc); ^1^H NMR (400 MHz, CDCl_3_) δ 6.90 (d, *J* = 7.2 Hz, 1H), 6.76–6.71 (m, 1H), 5.89–5.82 (m, 1H), 5.41 (dd, *J* = 15.6, 8.3 Hz, 1H), 4.54–4.43 (m, 1H), 4.39–4.29 (m, 2H), 4.11 (t, *J* = 6.0 Hz, 2H), 3.92 (dd, *J* = 8.4, 3.9 Hz, 2H), 3.72 (t, *J* = 5.6 Hz, 2H), 3.46 (s, *J* = 6.6 Hz, 3H), 3.32–3.22 (m, 2H), 2.37–2.24 (m, 3H), 1.92–1.72 (m, 8H), 1.43 (d, *J* = 1.4 Hz, 6H), 1.04 (s, 9H). ^13^C NMR (101 MHz, CDCl_3_) δ 171.58, 170.60, 148.47, 132.42, 130.08, 128.29, 121.68, 109.00, 81.76, 79.33, 79.15, 71.25, 61.32, 59.13, 48.55, 43.22, 36.20, 33.25, 29.71, 29.27, 29.05, 28.66, 27.27, 26.93, 25.72, 25.42, 22.70, 17.91, 14.13; HRMS (H-ESI) *m/z*: [M + H]^+^ calculated for C_28_H_49_N_2_O_8_ 541.3489; found 541.3485.

**Bengamide analogue 10.** Acetal **25** (12 mg, 0.022 mmol) was treated with 1 M HCl in exactly the same way as described above for the synthesis of this compound from **21**, to yield bengamide analogue **10** (9.1 mg, 83%), whose spectroscopic data were identical with those exhibited by **10** obtained above.

### 3.3. Cell Culture

The T84, SW480, HCT15, HT29 and MC38 colon cancer cell lines were selected to assay the bengamide effect. A normal colon fibroblast cell line (CCD18) was included in the analysis. In addition, a cell line of another tumor type (breast cancer) was studied (MCF7). All cell lines were grown in Dulbecco’s modified Eagle’s medium (DMEM) supplemented with fetal bovine serum (10%) and a mix of penicillin-streptomycin (1%) (Sigma-Aldrich, Madrid, Spain). Cell lines were maintained at 37 °C and 5% CO_2_ in a humidified incubator. 

### 3.4. Proliferation Assay 

The different cell lines were seeded in 48-well plates at a cell density of 3 × 10^3^ cells/well for MC38, 5 × 10^3^ cells/well for T84, SW480, HCT15 and CCD18, 15 × 10^3^ cells/well for HT29 and 4 × 10^3^ cells/well for MCF7. After attaching to the well, the treatments, Ben I and Ben V (analogues **5** and **10**, respectively), were added in a wide range of doses determined by the cell line from an initial stock of 0.3 mg/mL for Ben I and 6.9 mg/mL for Ben V, both dissolved in water. The treatments were maintained for 72 h. Elapsed this time, the cells were fixed with trichloroacetic acid at 10%, stained with Sulforhodamine B (Sigma-Aldrich, Madrid, Spain) and the dye was lifted with Trizma^®^ (Sigma-Aldrich, Madrid, Spain) at 10% following a previously established protocol [40]. Doxorubicin and 5-Fluorouracil were used as reference compounds in breast and colon cell lines, respectively. 

### 3.5. Hemocytotoxicity Assays

To evaluate the effect of Ben I and Ben V compounds on blood cells, a hemolysis test using human erythrocytes and a toxicity test using WBC were carried out. Different blood cells (erythrocytes and WBC) were isolated from blood samples taken from healthy donors and exposed to a wide range of doses of both Ben I and Ben V compounds (0.01–40 µM) following our previously described protocol [41]. Briefly, the erythrocytes were diluted 50 times their initial concentration in PBS, seeded in conical bottom 96-well plates and treated with the different treatments for 1 h at 37 °C under stirring. Following this, the plate was centrifuged, 100 µL of the supernatant was transferred to a new 96-well flat-bottom plate and the absorbance at 492 nm was determined. Erythrocytes treated with Triton X-100 to 20% were used as a positive control and as a negative control, erythrocytes treated with PBS were used. In the case of erythrocytes, the percentage of hemolysis (%HR) was calculated with the following formula:

$$\%HR=\frac{Abs\left(sample\right)-Abs\left(-control\right)}{Abs\left(+control\right)-Abs\left(-control\right)}\times 100.$$

After treatment, samples were observed with a light microscope and photographed (Leica DM IL LED).

As for the WBCs, they were seeded in flat-bottom 96-well plates in RPMI-1640 medium supplemented with FBS (10%) and a mixture of antibiotic (1%) at a density of 2 × 10^4^ cells/well. Treatments were added for 1 h and 12 h, after which the Cell Counting Kit-8 (CCK-8) (Dojindo Laboratories, Kumamoto, Japan) was used to determine viability. The CCK-8 reagent was added to each well and after 3 h of incubation the absorbance was measured at 450 and 620 nm to determine the viability according to the manufacturer.

### 3.6. Cell Cycle Assay

To assess the effect of treatments in the cell cycle, a cancer cell line (T84) and and a normal cell line (CCD18) were seeded in 6-well plates at a density of 1.5 × 10^5^ and 1 × 10^5^ cells/well, respectively. After 24 h, different concentrations of Ben I and Ben V (IC_25_ and IC_50_ at 48 h) were added and incubated at 37 °C and 5% CO_2_ for 48 h. In addition, untreated cells were used as a negative control and cells treated with 5-FU at its IC_50_ at 48 h as a positive control. After this time, the culture medium of each treatment was collected in a clean cytometry tube, the wells were washed with PBS at 4 °C and this wash was added to the previous tube. The cells were detached with a trypsin-EDTA mixture and also added to the tube. Next, the tubes were centrifuged at 3500 rpm for 3 min, the supernatant was discarded, and the pellet was resuspended in 100 µL of PBS. After this, 900 µL of 70% cold EtOH was added to each tube while the tubes were vortexed, then the tubes were placed on ice and incubated at 4 °C for 10 min. After this time, two washes were carried out with PBS to remove ethanol and the pellets were resuspended in 250 µL of PBS, to which 250 µL of DNA extraction solution was added (192 mL of 0.2 M Na_2_HPO_4_ + 8 mL of 0.1 M citric acid, pH = 7.8). The samples were incubated at 37 °C for 10 min, centrifuged and the supernatant discarded. Finally, 250 µL of propidium iodide (PI)/RNAse (ImmunoStep, Salamanca, Spain) solution was added and the samples were incubated for 10 min at 37 °C in the dark. Thereafter, the samples were analyzed by FAC Scan on a Becton Dickinson analyzer cytometer (Becton Dickinson, Franklin Lakes, NJ, USA).

## 4. Conclusions

In conclusion, the synthesized bengamide analogues are presented as a promising option for the treatment of CRC due to their great biocompatibility with blood cells in addition to the cells of the immune system (macrophages and WBC). Their greater antiproliferative effect on tumor cell lines than normal cells could be exploited to minimize the side effects caused by other chemotherapeutic agents. Further research in the future will be necessary to use this new therapeutic strategy in CRC.

## Figures and Tables

**Table 1:** Determination of IC_50_ (μM) ^(**a**)^ of Ben I (**5**) and Ben V (**10**) in tumor and non-tumor cell lines.

Cell Line	Ben I	Ben V	Reference Compound ^(b)^
CCD18	4.70 ± 0.28	5.08 ± 0.39	7.35 ± 0.41
T84	0.36 ± 0.03	0.07 ± 0.02	2.68 ± 0.16
SW480	0.59 ± 0.07	0.08 ± 0.00	6.35 ± 0.54
HCT15	1.90 ± 0.15	2.44 ± 0.25	6.58 ± 0.35
HT29	1.02 ± 0.19	0.66 ± 0.18	6.14 ± 0.94
MC38	1.60 ± 0.13	6.51 ± 1.12	0.33 ± 0.01
MCF7	0.49 ± 0.05	0.13 ± 0.01	0.04 ± 0.01

(**a**) Half-maximal inhibitory concentration (IC_50_) values calculated from dose-response curves as the concentration of compound that inhibits cell survival by 50% compared to control. They are expressed as means ± SD of triplicate samples each; (**b**) The reference compounds were doxorubicin for MCF7 and 5-fluorouracil for the rest of the cell lines (colon cell lines).

## Supplementary Materials

The following are available online at https://www.mdpi.com/1660-3397/18/5/240/s1. Figures S2–S6: 1H and 13C NMR Spectra of compounds.
