# Supplementary material for: Bengamide Analogues Show A Potent Antitumor Activity against Colon Cancer Cells: A Preliminary Study
Source: Mar Drugs. 2020 May 2;18(5):240. doi: 10.3390/md18050240 (PMC7281506; doi:10.3390/md18050240)
Supplement: Supplementary file 1 [file marinedrugs-18-00240-s001.pdf]

Supporting Information

Bengamide Analogues Show a Potent Antitumor Activity against Colon Cancer Cells: A Preliminary Study

Beatriz García-Pinel[1,2,3], Cristina Porras-Alcalá[4], Laura Cabeza[1,2,3,*], Raul Ortiz[1,2,3], José Prados[1,2,3], Consolación Melguizo[1,2,3], Iván Cheng-Sánchez[4], J. Manuel López-Romero[4,*], Francisco Sarabia[4,*]

[1] Institute of Biopathology and Regenerative Medicine (IBIMER), Center of Biomedical Research (CIBM), University of Granada, 18100 Granada, Spain
[2] Department of Anatomy and Embriology, Faculty of Medicine, University of Granada, 18071 Granada, Spain
[3] Instituto de Investigación Biosanitaria ibs. Granada, 18012 Granada, Spain
[4] Department of Organic Chemistry, Faculty of Sciences, University of Malaga, 29071 Málaga, Spain

* Correspondence: frsarabia@uma.es (F.S.); jmromero@uma.es (J.M.L.-R.); lautea@ugr.es (L.C.); Tel.: +34-952-134-258.

Index: Pages

^{1}H and ^{13}C NMR Spectra of compounds: S2-S6

^{1}H and ^{13}C NMR Spectra

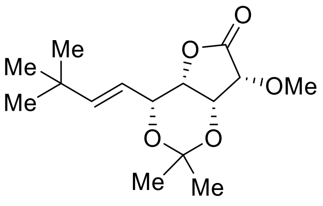

18

^{1}H NMR (400 MHz, $CDCl_3$)

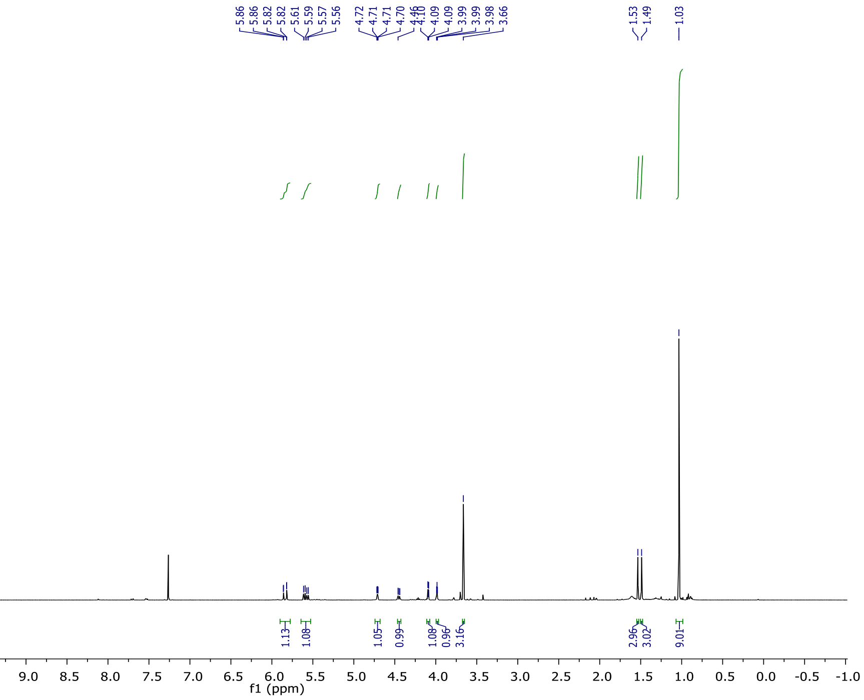

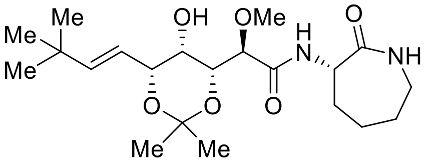

19

^{1}H NMR (400 MHz, $CDCl_3$)

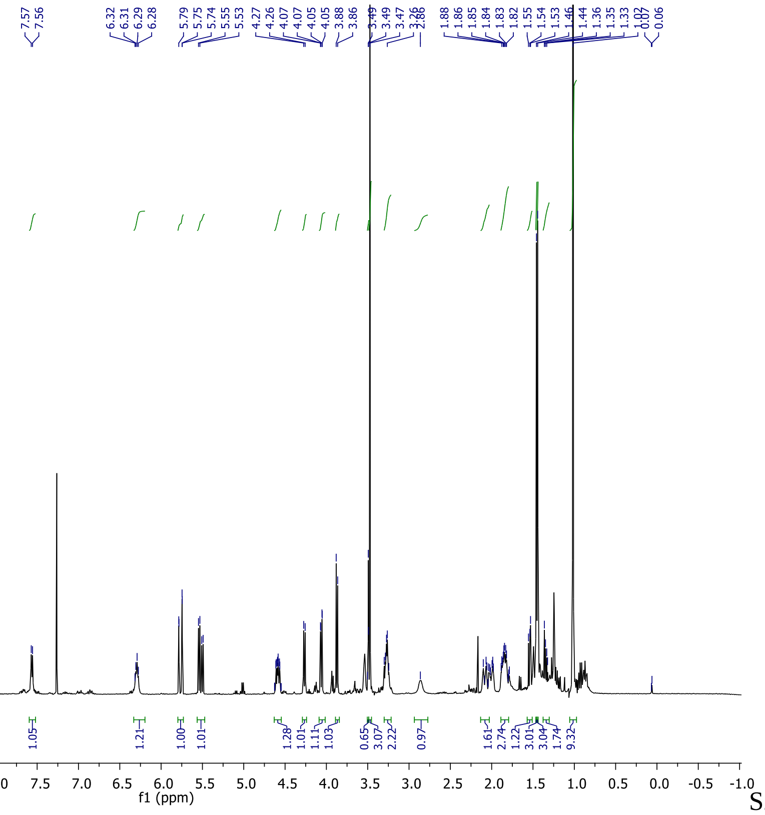

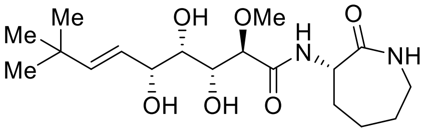

Ben I (**5**)

^{1}H NMR (400 MHz, $CDCl_3$)

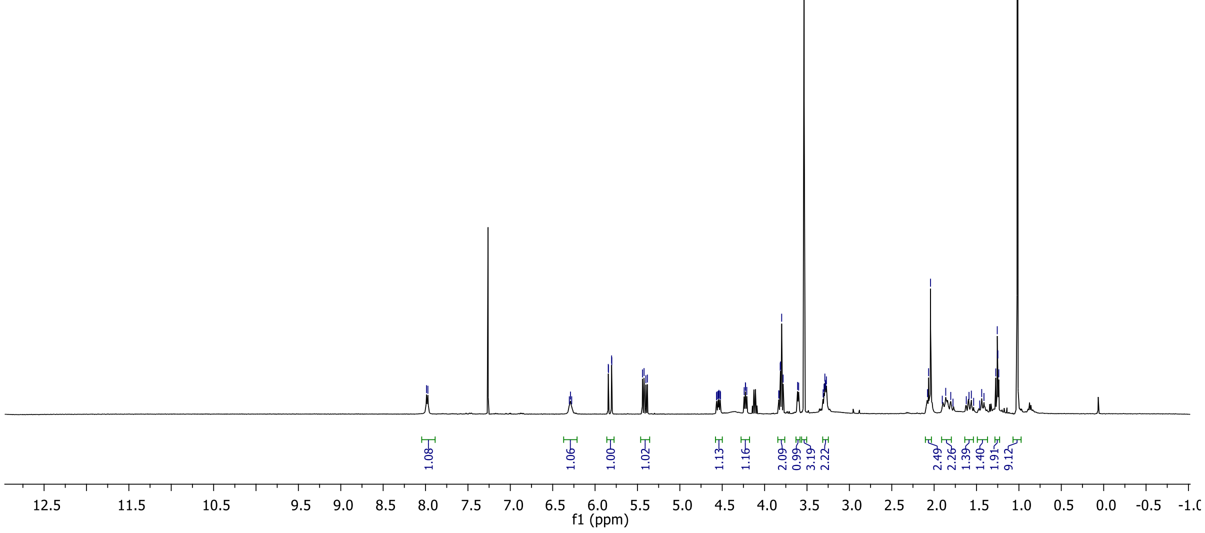

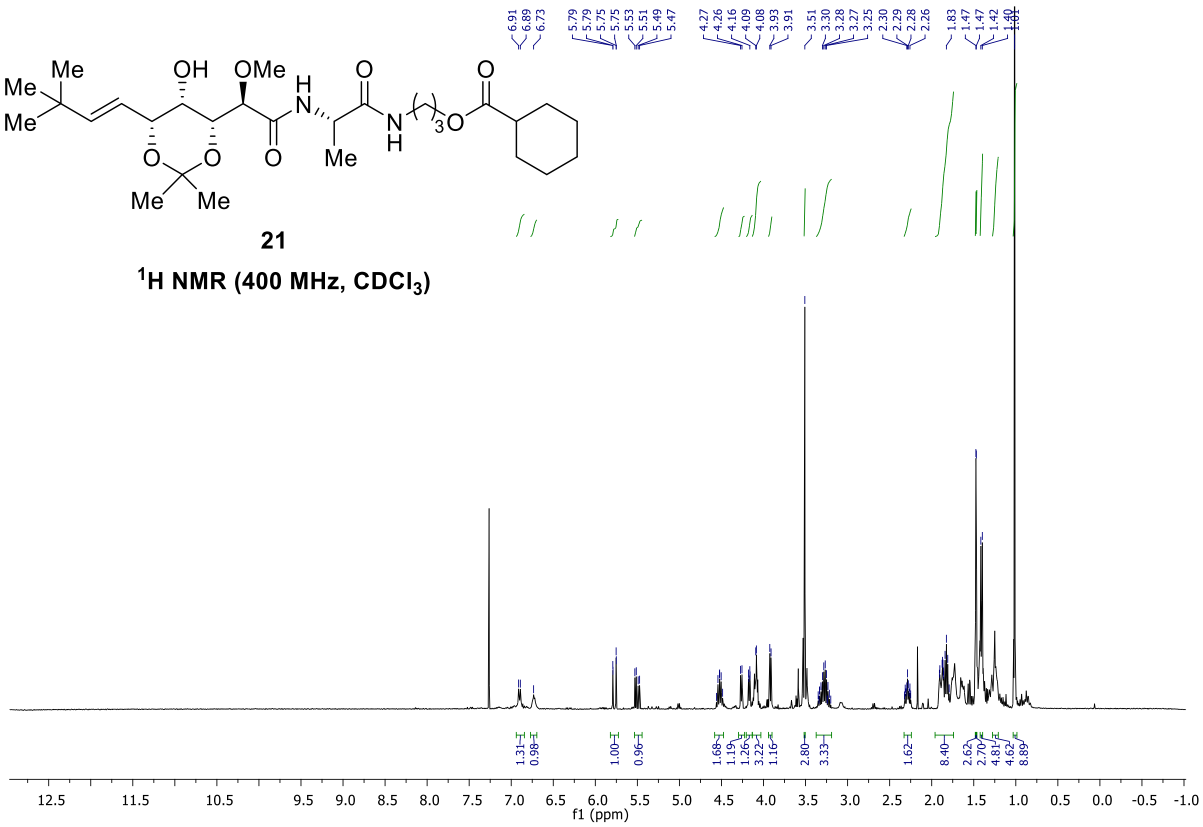

21

^{1}H NMR (400 MHz, CDCl$_3$)

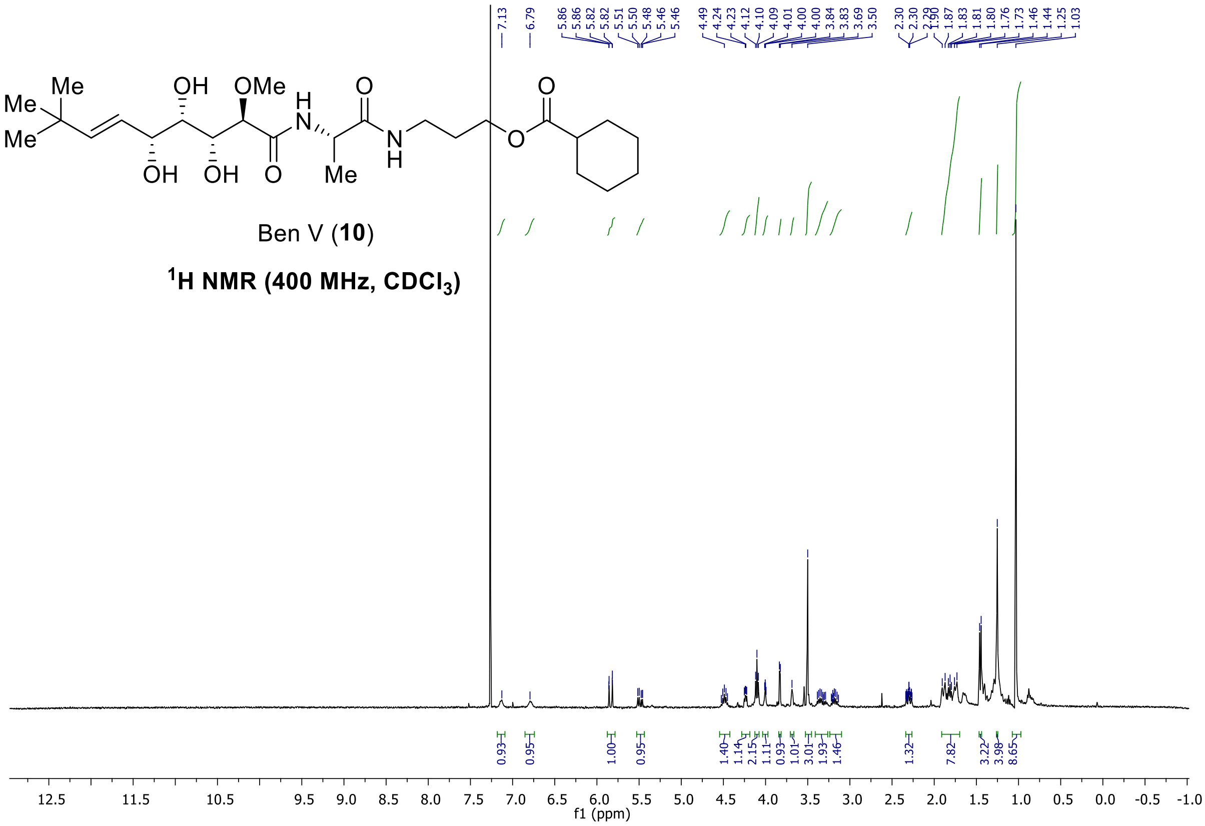

Ben V (**10**)

^{1}H NMR (400 MHz, CDCl$_3$)

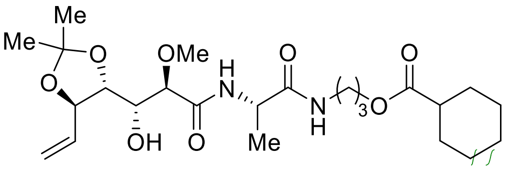

24

^{1}H NMR (400 MHz, CDCl$_3$)

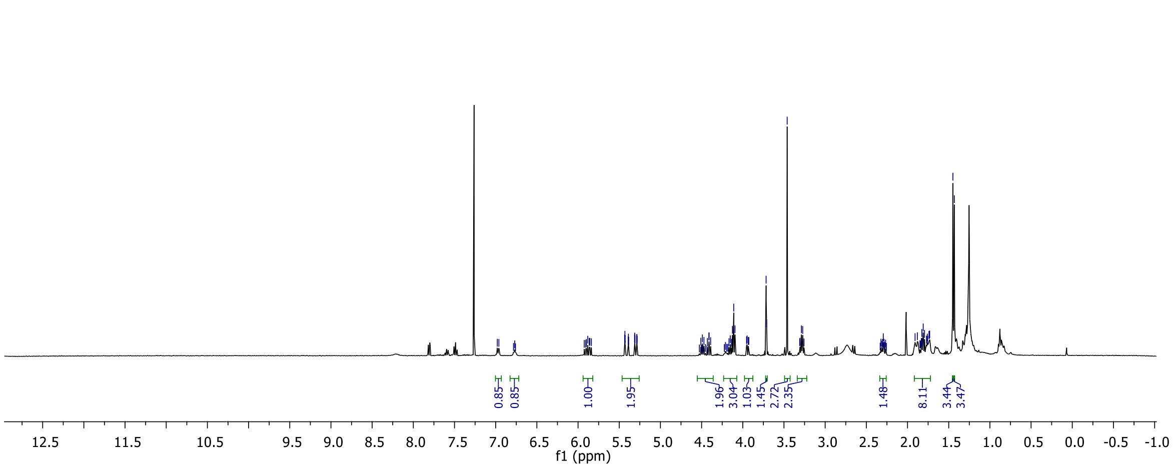

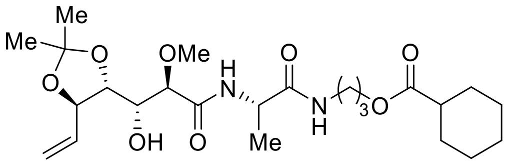

24

^{13}C NMR (100 MHz, CDCl$_3$)

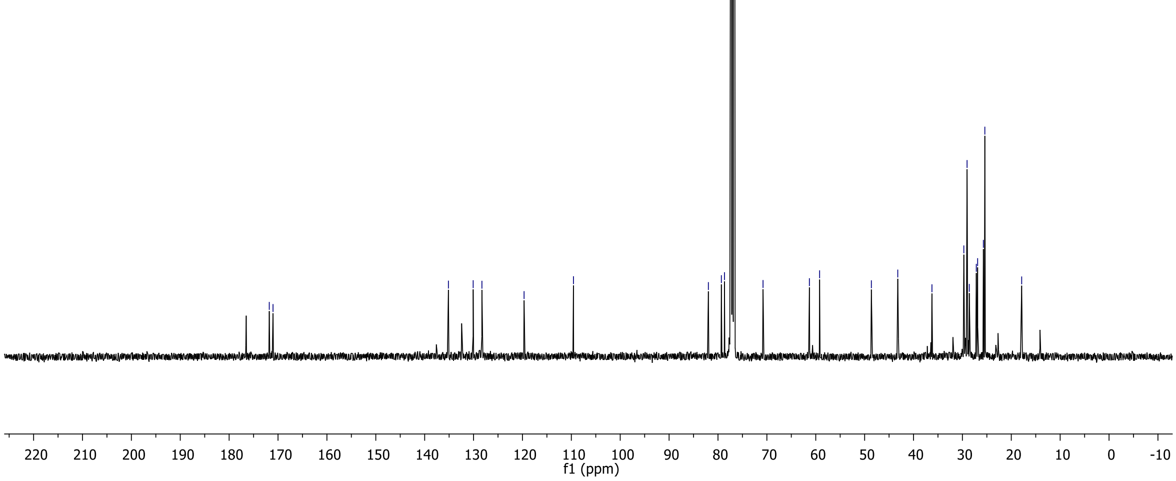

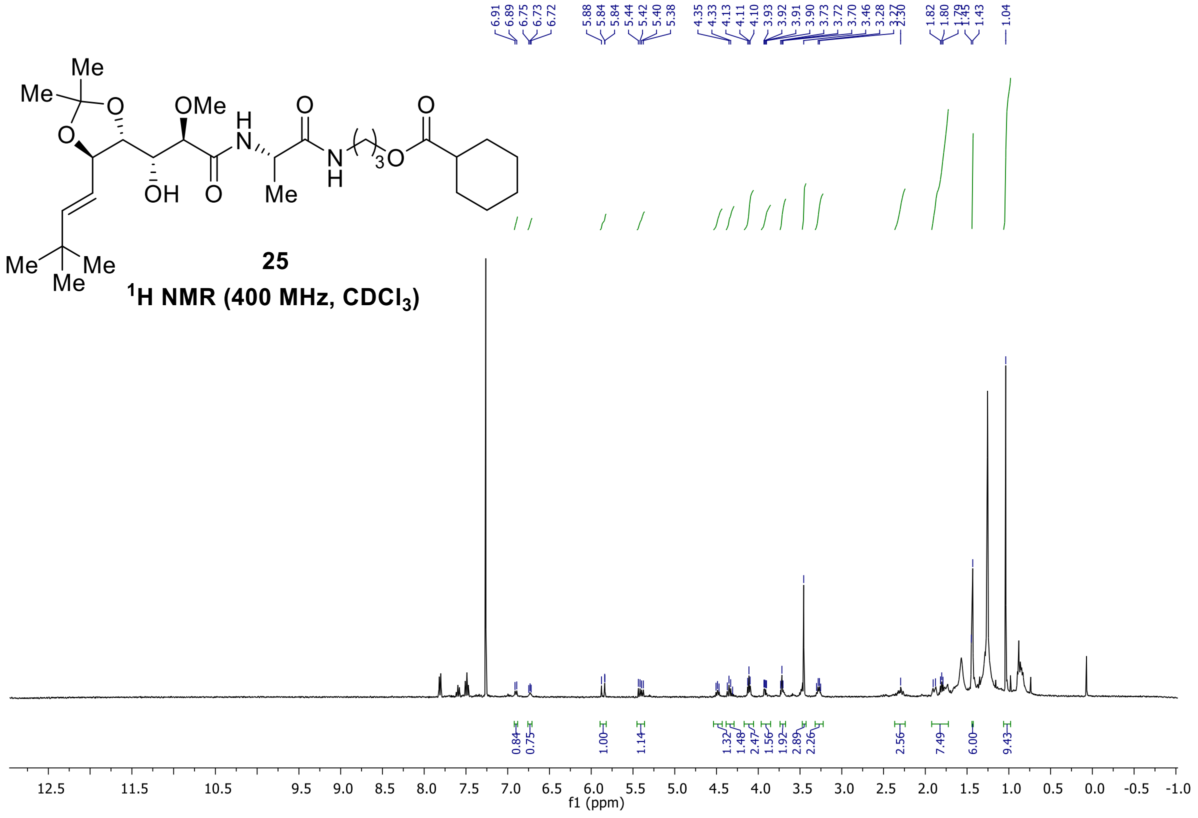
25
1H NMR (400 MHz, CDCl3)
f1 (ppm)

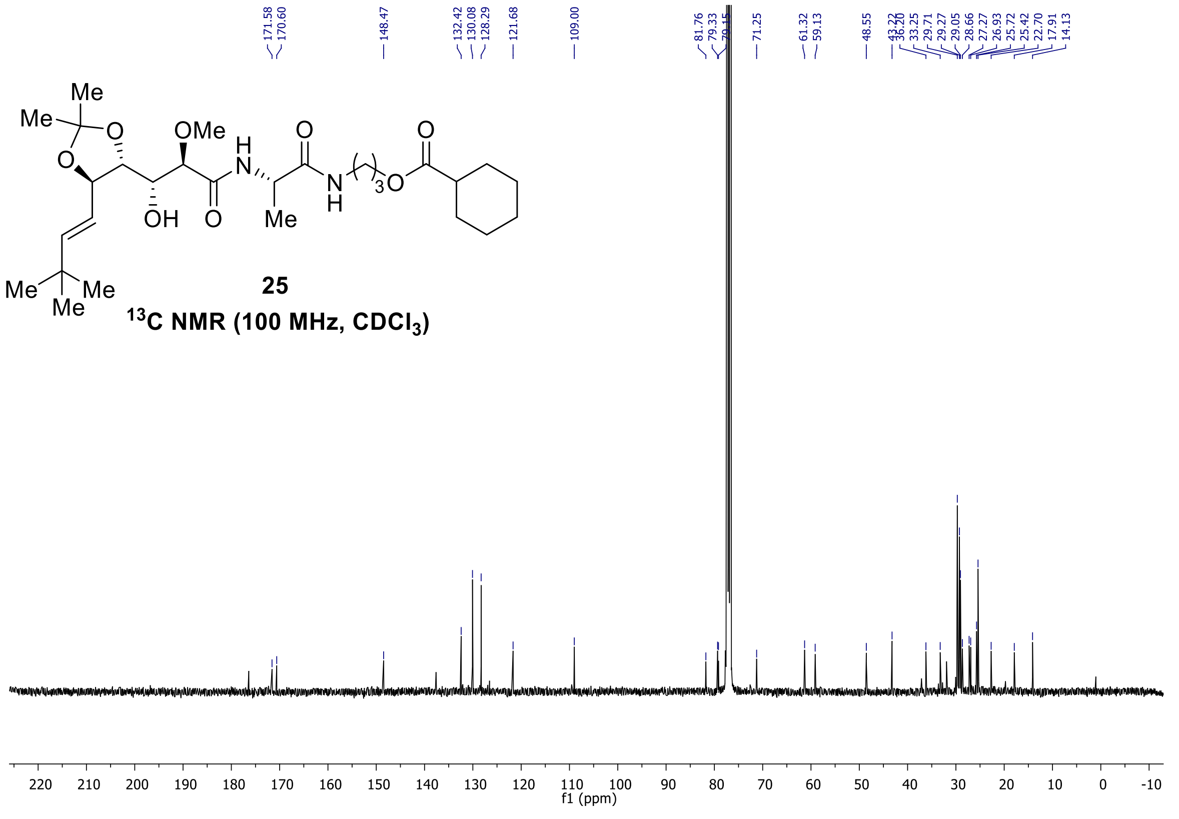
25
13C NMR (100 MHz, CDCl3)
f1 (ppm)
